# Patient‐Reported Outcomes and Surgical Results of Hand‐Sewn Versus Stapled Anastomosis for Lower Rectal Cancer Located 4–5 cm From the Anal Verge: A Subanalysis of the Ultimate Study

**DOI:** 10.1002/ags3.70063

**Published:** 2025-07-09

**Authors:** Masakatsu Numata, Jun Watanabe, Yuichiro Tsukada, Yusuke Suwa, Yosuke Fukunaga, Yasumitsu Hirano, Kazuhiro Sakamoto, Hiroki Hamamoto, Masanori Yoshimitsu, Hisanaga Horie, Nobuhisa Matsuhashi, Yoshiaki Kuriu, Shuntaro Nagai, Madoka Hamada, Shinichi Yoshioka, Shinobu Ohnuma, Tamuro Hayama, Koki Otsuka, Yusuke Inoue, Kazuki Ueda, Yuji Toiyama, Satoshi Maruyama, Shigeki Yamaguchi, Keitaro Tanaka, Takeshi Naitoh, Masahiko Watanabe, Motoko Suzuki, Toshihiro Misumi, Masaaki Ito, Ultimate Trial Group in Japan Society of Laparoscopic Colorectal Surgery

**Affiliations:** ^1^ Department of Surgery, Gastroenterological Center Yokohama City University Medical Center Kanagawa Japan; ^2^ Department of Colorectal Surgery Kansai Medical University Osaka Japan; ^3^ Department of Colorectal Surgery National Cancer Center Hospital East Chiba Japan; ^4^ Department of Gastroenterological Surgery Cancer Institute Hospital, Japanese Foundation of Cancer Research Tokyo Japan; ^5^ Division of Gastroenterological Surgery Saitama Medical University International Medical Center Saitama Japan; ^6^ Department of Coloproctological Surgery Juntendo University Faculty of Medicine Tokyo Japan; ^7^ Department of General and Gastroenterological Surgery Osaka Medical and Pharmaceutical University Osaka Japan; ^8^ Department of Surgery Hiroshima City North Medical Center Asa Citizens Hospital Hiroshima Japan; ^9^ Department of Surgery Jichi Medical University Tochigi Japan; ^10^ Department of Gastroenterological surgery and Pediatric surgery Gifu University, Graduate School of Medicine Gifu Japan; ^11^ Department of Surgery Kyoto Prefectural University of Medicine Kyoto Japan; ^12^ Department of Surgery and Oncology Graduate School of Medical Sciences, Kyushu University Fukuoka Japan; ^13^ Digestive Disease Center Chikamori Hospital Kochi Japan; ^14^ Department of Surgery Yao Municipal Hospital Osaka Japan; ^15^ Department of Surgery Tohoku University Hospital Miyagi Japan; ^16^ Department of Surgery Teikyo University school of medicine Tokyo Japan; ^17^ Department of Surgery Iwate Medical University School of Medicine Iwate Japan; ^18^ Department of Surgery Nagasaki University Graduate School of Biomedical Sciences Nagasaki Japan; ^19^ Division of Endoscopic & Colorectal Surgery, Department of Surgery Kindai University, Faculty of Medicine Osaka Japan; ^20^ Department of Gastrointestinal and Pediatric Surgery Mie University Mie Japan; ^21^ Department of Gastroenterological Surgery Niigata Cancer Center Hospital Niigata Japan; ^22^ Division of Colorectal Surgery, Department of Surgery Tokyo Women's Medical University Tokyo Japan; ^23^ Department of General, Breast and Digestive Surgery Otsu City hospital Shiga Japan; ^24^ Department of Lower Gastrointestinal Surgery Kitasato University School of Medicine Kanagawa Japan; ^25^ Department of Surgery Kitasato University Kitasato Institute Hospital Tokyo Japan; ^26^ Department of Data Science National Cancer Center Hospital East Chiba Japan

**Keywords:** anorectal function, hand‐sewn anastomosis, lower anterior resection, rectal cancer

## Abstract

**Background:**

Preserving anorectal function while achieving oncological success is crucial in the treatment of lower rectal cancer near the anal canal. Despite advancements in laparoscopic surgery that facilitate anal preservation, post‐operative anorectal dysfunction considerably affects quality of life. Both hand‐sewn and stapled anastomoses are suitable options for tumors located 4–5 cm from the anus. However, evidence comparing the functional outcomes and complications associated with both anastomosis methods is lacking.

**Methods:**

This multicenter, single‐arm prospective study included patients with cT1‐T2/N0/M0 adenocarcinoma located 4–5 cm from the anal verge, scheduled for upfront laparoscopic surgery. Anorectal function, post‐operative complications, urinary and male sexual function, and oncological outcomes were assessed using the validated scores.

**Results:**

A total of 135 patients were analyzed and divided into hand‐sewn (*n* = 65) and stapled (*n* = 70) groups. The patient characteristics were similar, except for the tumors in the hand‐sewn group located 1 mm closer to the anal verge. No significant differences were observed in the post‐operative complications. Anorectal function, measured using Wexner scores, worsened at 3 months postoperatively and gradually improved in both groups. At 3, 6, 12, 24, and 36 months, the stapled group consistently showed better Wexner scores than the hand‐sewn group. Urinary function, sexual function, and oncological outcomes were similar in both groups.

**Conclusion:**

Stapled anastomosis may provide better anorectal function with comparable safety and oncological outcomes to hand‐sewn anastomosis. Therefore, stapled anastomosis may be preferred for tumors located 4–5 cm from the anal verge to ensure oncological safety.

**Trial Registration:**

This study was registered in the UMIN Clinical Trials Registry System (UMIN 000011750)

## Graphical abstract

Preserving anorectal function while ensuring oncological success is critical in lower rectal cancer surgery. This multicenter prospective study compared hand‐sewn and stapled anastomosis in 135 patients with tumors 4–5 cm from the anal verge. Although post‐operative complications and oncological outcomes were similar, stapled anastomosis consistently demonstrated better anorectal function.

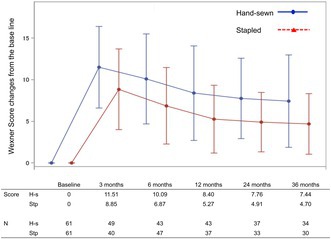

## 1 Introduction

Colorectal cancer (CRC) is the third most prevalent cancer worldwide, with more than 30% of cases originating in the rectum [1, 2]. Preservation of anorectal function and oncological outcomes are crucial in the management of lower rectal cancer near the anal canal. The development of laparoscopic surgery has enabled elaborate dissection around the deep pelvic area, facilitating anal preservation even in cases of ultralow rectal cancer, without compromising curability. However, one of the major post‐operative complaints is anorectal dysfunction, which considerably affects patient's quality of life (QOL) [3]. Moreover, other complications such as anastomotic leakage and urinary and sexual dysfunctions further contribute to the deterioration of QOL in patients who undergo surgery for low rectal cancer surgery [4, 5, 6].

Two primary anastomosis techniques are widely established for lower rectal cancer near the anal canal: the first technique is the stapled anastomosis, where the rectum is transected just above the anal canal from the abdominal side using an automatic stapler, and subsequently, anastomosis is performed using an automatic anastomosis stapler [7]. The second is hand‐sewn anastomosis, which involves transection of the rectum from the anal side, follow by dissection of the intersphincteric plane with subsequent hand‐sewn anastomosis [8]. Generally, transection from the abdominal side becomes more challenging when the tumor is located closer to the anus. Consequently, hand‐sewn anastomosis tends to be preferred over stapled anastomosis when the tumor is located closer to the anus.

This study is a subanalysis of a previously reported Ultimate trial [9]. According to the main analysis of the Ultimate trial, the choice of anastomosis technique varies depending on the distance from the anal verge to the tumor. For patients with a distance of up to 4 cm from the anal verge to the tumor, the selection rate for stapled anastomosis was 22.3%. However, for patients with a distance of 4–5 cm, the selection rate for the same increased to 50.7%, while hand sewing was chosen in 47.1% of cases. Thus, for patients with a distance of 4–5 cm from the anal verge, both hand sewing and stapling may be feasible.

Several studies have compared both methods, and many of these reports suggest superior anal function outcomes with stapled than with hand‐sewn anastomosis [7, 10, 11]. However, evidence comparing all short‐term complications, anorectal function, urinary function, sexual function, and oncologic outcomes in a prospective setting is lacking.

Thus, we comprehensively compared the outcomes of hand‐sewn and stapled anastomoses at tumor‐to‐anal verge distances of 4–5 cm, focusing on patient‐reported anorectal function.

## 2 Materials and Methods

### 2.1 Study Design and Participants

This study presents a secondary analysis of the data obtained in the Ultimate study [9]. All eligible patients from 47 specialized Japanese hospitals affiliated with the Japan Society of Laparoscopic Colorectal Surgery were prospectively registered. The eligibility and exclusion criteria for this study are as documented in the initial report [9]: the key eligibility criteria were a clinical diagnosis of T1‐T2/N0/M0 adenocarcinoma, located within 5 cm from the anal verge or 3 cm from the dentate line, and scheduled upfront laparoscopic surgery with curative intent.

### 2.2 Baseline Tumor Staging

All patients underwent tumor staging through physical examination, total colonoscopy, computed tomography, and pelvic magnetic resonance imaging, according to UICC TNM staging manual 8th edition [12]. The distance between the tumor and anal verge was recorded via digital examination and colonoscopy.

### 2.3 Surgical Procedure

All surgical interventions were performed laparoscopically. According to the study protocol, only surgeons with an experience of a minimum of 30 laparoscopic rectal cancer surgeries were eligible. For tumor located 5 cm from the anal verge, transabdominal total mesorectal excision involves dissecting the tumor down to the distal side. The procedure for dissecting beyond the tumor toward the anal side was the same for both techniques. At this stage, both hand‐sewn and stapled anastomoses are theoretically possible, and the choice between them is at the surgeon's discretion. For hand‐sewn cases, a transanal approach was adopted after total mesenteric excision. Circumferential mucosal and muscular incisions were made at least 1 cm below the tumor margin, followed by rectal closure using the purse‐string suture technique. Intersphincteric dissection between the puborectalis and internal sphincters was meticulously performed, and the specimen was extracted. After sufficient intrapelvic irrigation to prevent tumor implantation, hand‐sewn end‐to‐end coloanal anastomosis was performed. For stapled cases, rectal transection was performed using an automated linear stapler with an adequate distal margin. Subsequently, coloanal anastomosis was performed using a circular stapler and double‐stapling technique. The creation of a diverting stoma was at the surgeon's discretion.

### 2.4 Post‐Operative Outcome

Post‐operative outcomes were divided into post‐operative course and post‐operative complications (Clavien–Dindo classification grade III or higher). Post‐operative complications were further categorized into early post‐operative complications, occurring from the end of surgery to 1 month postoperatively, and late post‐operative complications, occurring from 1 month to 3 years postoperatively.

### 2.5 Anorectal Function Assessment

An assessment of post‐operative anorectal function was conducted using the Wexner score [13]. The Wexner score, which ranges from 0 to 20, with higher scores indicating poorer function, consists of five items regarding continence for solid and liquid stools, gas, pad usage, and lifestyle alterations. Patients without temporary diverting stoma were evaluated at six intervals: preoperatively and at 3, 6, 12, 24, and 36 months postoperatively. Patients with temporary diverting stomas were evaluated at six intervals: preoperatively and at 3, 6, 12, 24, and 36 months after stoma closure.

### 2.6 Urinary Function Assessment

Urinary function was assessed preoperatively and at 1 week, 1 month, and 3, 6, and 12 months postoperatively, totaling six assessments, using three questionnaires: the International Prostate Symptom Score (IPSS) [14], Overactive Bladder Symptom Score (OABSS) [15], and International Consultation on Incontinence Questionnaire‐Short Form (ICIQ‐SF) [16].

### 2.7 Male Sexual Function Assessment

Utilizing the International Index of Erectile Function (IIEF)‐15 and its condensed 5‐item version (IIEF‐5), male sexual function was evaluated in patients ≤ 70 years. The assessment involved administering questionnaires four times: preoperatively and 3, 6, and 12 months postoperatively. While the IIEF‐5 primarily focuses on erectile function, the IIEF‐15 provides a more comprehensive assessment that includes aspects of sexual satisfaction [17].

### 2.8 Statistical Analysis

Categorical data are described as proportions, and comparisons were made using the chi‐square test. The correlation between two numerical variables was evaluated using a *t*‐test. For the anorectal, urinary, and male sexual function assessments, a descriptive analysis of the quantitative data was conducted using the mean and standard deviation. Recurrence‐free and overall survival curves were plotted using the Kaplan–Meier method, and differences between the two groups were assessed using the log‐rank test. Cumulative local recurrence was assessed using Gray's test. Statistical significance was set at *p* < 0.05. All the analyses were performed using SAS version 9.4 (SAS Institute Inc., Cary, NC, USA).

## 3 Results

### 3.1 Patient Enrolment

**FIGURE 1:**
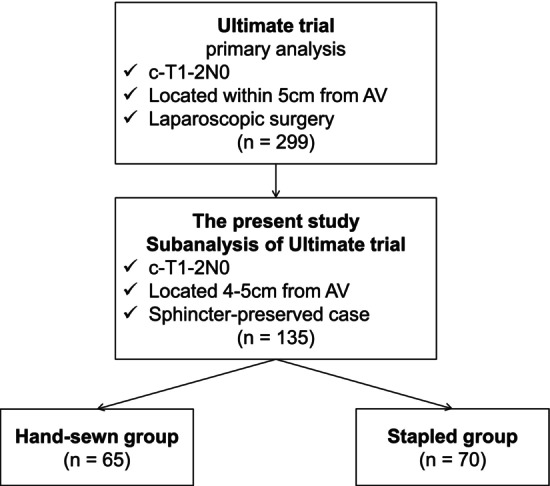
Patient flow diagram. AV, anal verge.

In the primary Ultimate trial [9], a total of 300 patients were registered, and 299 were analyzed between January 2014 and March 2017. From these, data of patients with tumors located 4–5 cm from the anal verge were included in the subanalysis (*n* = 138). After excluding three non‐sphincter‐preserved cases, 135 sphincter‐preserved cases were divided into hand‐sewn and stapled groups (*n* = group, *n* = 65 and 70, respectively) (Figure 1).

### 3.2 Patient Characteristics

The patient characteristics of both groups are summarized in Table 1. No statistically significant differences were observed between the two groups except for the distance from the anal verge to the tumor (hand‐sewn: 48.7 mm vs. stapled: 49.5 mm, *p* = 0.01). Conversely, no significant distinction was noted in the distance from the dentate line to the tumor (hand‐sewn: 28.8 mm vs. stapled: 28.5 mm, *p* = 0.78). The hand‐sewn group had 53.8% cT1 and 46.2% cT2, while the stapled group had 64.3% cT1 and 35.7% cT2, with no significant differences between the groups. To further investigate the potential selection bias in anastomotic procedure, we conducted a subgroup analysis stratified by whether splenic flexure mobilization was performed. The background characteristics within each subgroup are summarized in Tables S1 (mobilized) and S2 (not mobilized). No statistically significant differences were observed in most baseline factors between the stapled and hand‐sewn groups in either subgroup, although the hand‐sewn group had a higher proportion of cT2 tumors when splenic flexure mobilization was not performed (*p* = 0.01).

**TABLE 1:** Patient characteristics.

	Hand‐sewn (*n* = 65)	Stapled (*n* = 70)	*p*
Age	63.6 (10.63)	62.3 (10.38)	0.46
Sex			
Male	50 (76.9%)	45 (64.3%)	0.11
Female	15 (23.1%)	25 (35.7%)	
ECOG‐PS			
0	61 (93.8%)	67 (95.7%)	0.62
1	4 (6.2%)	3 (4.3%)	
Abdominal Surgery History	11 (16.9%)	20 (28.6%)	0.11
BMI	22.9 (2.93)	23.2 (3.45)	0.62
Tumor distance from AV	48.7 (2.21)	49.5 (1.67)	0.01
Tumor distance from DL	28.8 (4.60)	28.5 (4.84)	0.78
CEA	2.9 (2.28)	2.8 (3.02)	0.82
CA19‐9	11.7 (11.77)	9.0 (7.38)	0.11
Tumor laterality			
Left	11 (16.9%)	12 (17.1%)	0.16
Right	6 (9.2%)	11 (15.7%)	
Anterior	28 (43.1%)	18 (25.7%)	
Posterior	20 (30.8%)	29 (41.4%)	
cT			
T1	35 (53.8%)	45 (64.3%)	0.22
T2	30 (46.2%)	25 (35.7%)	
cN			
N0	65 (100)	70 (100)	
Adjuvant chemotherapy	13 (20.0%)	10 (14.3%)	0.38
Time from primary surgery to initial anorectal functional assessment			
Months	9.4 (4.11)	6.6 (3.96)	0.0002

*Note:* Values are presented *n* (%) for categorical data, and mean with standard deviation.

Abbreviations: AV, anal verge; BMI, body mass index; CA19‐9, carbohydrate antigen 19‐9; CEA, carcinoembryonic antigen; DL, dentate line; ECOG‐PS, Eastern Cooperative Oncology Group Performance Status.

### 3.3 Operative Outcomes

The operative outcomes are summarized in Table 2. The operative time was similar in both groups (278.9 vs. 271.1 min, *p* = 0.63); however, blood loss appeared to be higher in the hand‐sewn group compared to the stapled group, though the difference did not reach statistical significance (61.1 vs. 37.5 mL, *p* = 0.05). Conversion to open surgery was not observed in either group. With regard to short‐term morbidity, including anastomotic leakage (10.8% vs. 8.6%, *p* = 0.67) and wound infection (6.2% vs. 8.1%, *p* = 0.82), there were no significant intergroup differences. Anastomotic stenosis was the most common complication (3.1% vs. 4.3%; *p* = 0.71). In terms of pathological findings (Table 2), lymph node harvesting was similar in both groups (12.4 vs. 12.8, *p* = 0.78). No differences were observed in pT, pN, or pStage between the groups. Both groups had comparable proximal margins (176.4 vs. 163.0 mm, *p* = 0.17), while a significant difference was noted in the distal margin (18.3 vs. 12.8 mm, *p* = 0.0001). Negative radial margins were observed in all patients.

**TABLE 2:** Operative and pathological outcomes.

	Hand‐sewn (*n* = 65)	Stapled (*n* = 70)	*p*
Operative time (min)	278.9 (104.46)	271.1 (82.17)	0.63
Blood loss (mL)	61.1 (64.35)	37.5 (77.61)	0.05
Lymph node dissection			
D2	16 (24.6%)	42 (60.0%)	0.00003
D3	49 (75.4%)	28 (40.0%)	
LCA preservation	17 (26.2%)	45 (64.3%)	0.000009
SF mobilization	33 (50.8%)	10 (14.3%)	0.000005
Conversion	0 (0.0%)	0 (0.0%)	
AN preservation			
Complete	64 (98.5%)	70 (100.0%)	0.30
Incomplete	1 (1.5%)	0 (0.0%)	
Diverting stoma			
None	1 (1.5%)	16 (22.9%)	0.0002
Ileostomy	64 (98.5%)	51 (72.9%)	
Colostomy	0 (0.0%)	3 (4.3%)	
Trans‐anal drain	22 (33.8%)	39 (55.7%)	0.01
Days to first flatus	1.2 (0.36)	1.4 (1.10)	0.07
Days to start drink	1.1 (0.45)	1.2 (0.61)	0.60
Re‐operation	2 (3.1%)	3 (4.3%)	0.71
Short‐term morbidity			
Anastomotic leakage	7 (10.8%)	6 (8.6%)	0.67
Wound infection	4 (6.2%)	5 (7.1%)	0.82
Ileus	0 (0.0%)	3 (4.3%)	0.09
Urinary infection	1 (1.5%)	1 (1.4%)	0.96
Late‐term morbidity			
Anastomotic stenosis	2 (3.1%)	3 (4.3%)	0.71
Anastomotic leakage	0 (0.0%)	1 (1.4%)	0.33
ileus	1 (1.5%)	0 (0.0%)	0.30
Pelvic abscess	1 (1.5%)	0 (0.0%)	0.30
Rectal prolapse	1 (1.5%)	0 (0.0%)	0.30
Tumor diameter (mm)	23.7 (11.92)	25.7 (13.97)	0.38
Lymph node harvest	12.4 (7.06)	12.8 (6.76)	0.78
pT			
Tis	2 (3.1%)	1 (1.4%)	0.14
T1	28 (43.1%)	44 (62.9%)	
T2	26 (40.0%)	18 (25.7%)	
T3	9 (13.8%)	7 (10.0%)	
pN			
N0	54 (83.1%)	58 (82.9%)	0.77
N1a	5 (7.7%)	6 (8.6%)	
N1b	6 (9.2%)	5 (7.1%)	
N2a	0 (0.0%)	1 (1.4%)	
pStage			
0	2 (3.1%)	1 (1.4%)	0.90
I	46 (70.8%)	52 (74.3%)	
II	6 (9.2%)	5 (7.1%)	
IIIIA	8 (12.3%)	10 (14.3%)	
IIIB	3 (4.6%)	2 (2.9%)	
Proximal margin (mm)	176.4 (62.49)	163.0 (50.73)	0.17
Distal margin (mm)	18.3 (7.34)	12.8 (6.05)	0.0001
Radial margin			
Negative	65 (100.0%)	70 (100.0%)	

*Note:* Values are presented *n* (%) for categorical data, and mean with standard deviation.

Abbreviations: AN, autonomic nerve; LCA, left colic artery; SF, splenic flexure.

### 3.4 Anorectal Functional Assessment

The analysis included 122 cases (61 in each group) after excluding cases with unclosed diverting stomas and incomplete survey responses. Specifically, four patients were excluded from the hand‐sewn group due to incomplete survey responses. In the stapled group, nine patients were excluded—eight due to incomplete survey responses and one who did not undergo ileostomy closure at the patient's request.

**FIGURE 2:**
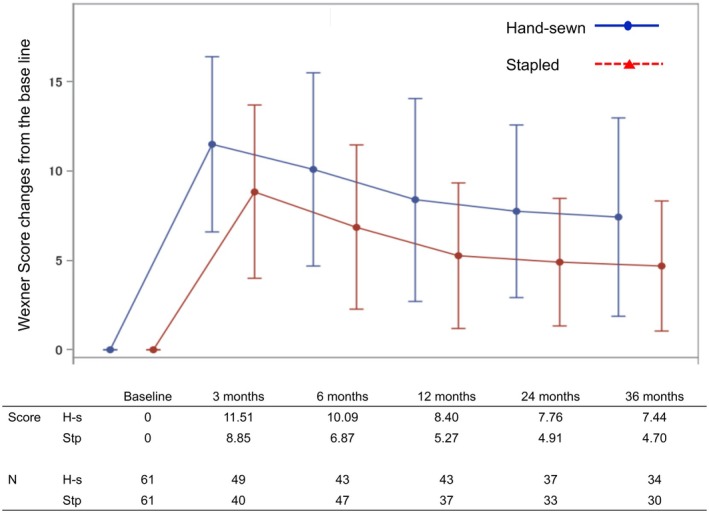
Anorectal function. H‐s, hand‐sewn; Stp, stapled.

The median duration from surgery to the initial anorectal function assessment was significantly longer in the hand‐sewn group compared to the stapled group (9.2 vs. 6.1 months, *p* = 0.0002). This reflects the higher proportion of patients with temporary diverting stomas in the hand‐sewn group, which delayed the onset of postoperative functional evaluation. Figure 2 shows the changes from baseline in Wexner scores at each assessment time point for both groups. The anorectal function in both groups worsened at 3 months postoperatively, followed by gradual improvement until 3 years postoperatively. The Wexner score changes from baseline in the hand‐sewn group at post‐operative 3, 6, 12, 24, and 36 months were 11.51, 10.09, 8.40, 7.76, and 7.44, respectively, while those in the stapled group were 8.85, 6.87, 5.27, 4.91, and 4.70, respectively. The score differences between the two groups at 3, 6, 12, 24, and 36 months postoperatively were 2.66, 3.22, 3.13, 2.85, and 2.74, respectively, with the stapled group consistently exhibiting a 2–3 points improvement at all evaluation points.

To further explore whether this trend was consistent across different case complexities, we performed a subgroup analysis of Wexner score changes stratified by the presence or absence of splenic flexure mobilization. The results are shown in Figures S1 (with mobilization) and S2 (without mobilization). In both subgroups, the stapled group consistently exhibited 2–3 point lower Wexner scores from baseline at each postoperative time point, reaffirming that the favorable continence outcomes associated with stapled anastomosis were observed regardless of surgical complexity.

We also conducted a separate subgroup analysis limited to patients who had undergone diverting ileostomy to assess whether the difference in evaluation timing influenced the results. Although this approach aimed to reduce the potential bias associated with the timing of postoperative anorectal function assessment, a significant difference remained between the groups in the time from surgery to initial evaluation (9.4 ± 4.11 months in the hand‐sewn group vs. 7.3 ± 3.98 months in the stapled group, *p* = 0.0067). Nonetheless, the stapled group continued to show lower Wexner scores at all postoperative time points in this subgroup analysis (Figure S3).

### 3.5 Urinary Functional Assessment

**FIGURE 3:**
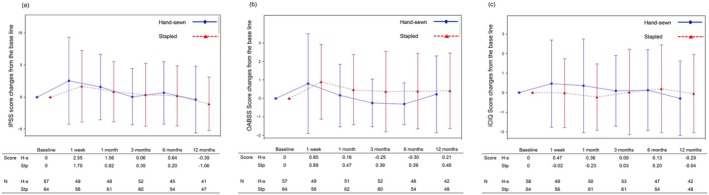
Urinary function. (a) IPSS. H‐s, hand‐sewn; IPSS, international prostate symptom score; Stp, stapled. (b) OABSS. H‐s, hand‐sewn; OABSS, overactive bladder symptom score; Stp, stapled. (c) ICIQ. H‐s, hand‐sewn; ICIQ, international consultation of incontinence questionnaire; Stp, stapled.

The results of the urinary functional assessment using the three questionnaires are shown in Figure 3. According to the evaluation using the IPSS, the scores increased from baseline at 1 week postoperatively; however, the change in scores decreased at 1 month postoperatively; by 3 months, the scores returned to near baseline levels. Evaluation using the OABSS and ICIQ‐SF did not reveal clinically significant changes from baseline at each assessment point. The difference between the groups in terms of the IPSS scores at each assessment point was 0.85, 0.74, −0.29, 0.44, and 0.67. Similarly, the difference in OABSS scores between the groups at each assessment point was −0.09, −0.31, −0.64, −0.69, and −0.19, while that for ICIQ‐SF scores was 0.49, 0.59, 0.06, −0.07, and −0.25. In all assessments using the IPSS, OABSS, and ICIQ‐SF, the values between the groups were similar and no clinically significant differences were observed.

### 3.6 Male Sexual Functional Assessment

**FIGURE 4:**
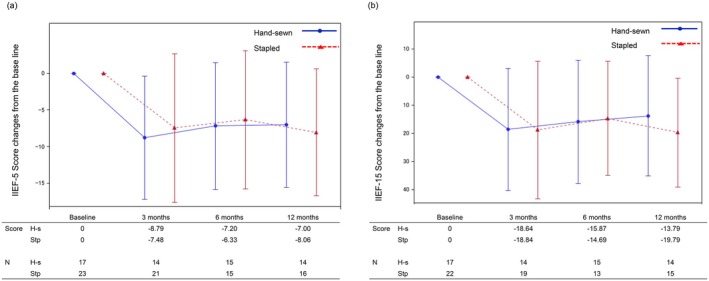
Male sexual function. (a) IIEF‐5. H‐s, hand‐sewn; IIEF‐5, international index of erectile function‐5; Stp, stapled. (b) IIEF‐15. H‐s, hand‐sewn; IIEF‐15, international index of erectile function‐15; Stp, stapled.

Male sexual function was evaluated using IIEF‐5 and IIEF‐15 (Figure 4). The scores for both IIEF‐5 and IIEF‐15 decreased compared to baseline at 3 months postoperatively, and there was no improvement at 6 and 12 months. The differences in IIEF‐5 change scores between the two groups were −1.31, −0.87, and 1.06 at 3, 6, and 12 months, respectively. Similarly, the difference in IIEF‐15 change scores between the two groups was 0.02, −1.18, and 6.00 at 3, 6, and 12 months, respectively. Both groups showed similar score changes in sexual function recovery with no consistent trends.

### 3.7 Oncological Outcome

**FIGURE 5:**
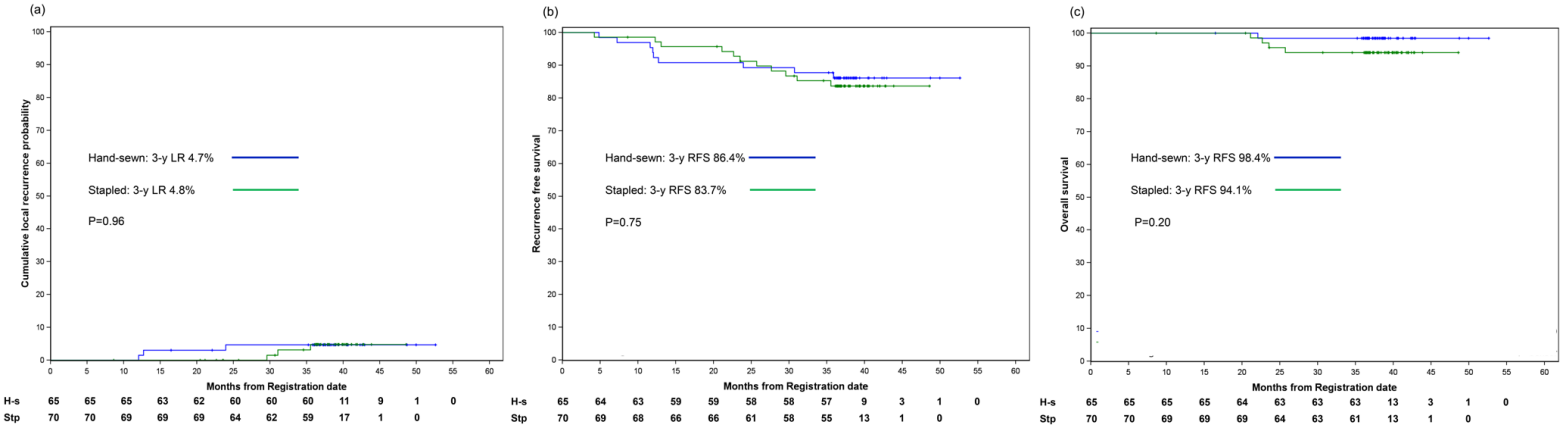
Oncological results. (a) Cumulative local recurrence. 3‐y LR, three‐year cumulative local recurrence rate. (b) Recurrence free survival. 3‐y RFS, three‐year recurrence free survival rate. (c) Overall survival. 3‐y OS, three‐year overall survival rate.

With a median follow‐up period of 36 months, the 3‐year cumulative locoregional recurrence rates were 4.7% and 4.8% in the hand‐sewn and stapled groups, respectively. The 3‐year recurrence‐free and overall survival rates for the hand‐sewn and stapled groups were 86.4% and 83.7% (*p* = 0.75) and 98.4% and 94.1% (*p* = 0.20), respectively. Consequently, no significant differences in oncological outcomes were observed between the two groups (Figure 5).

## 4 Discussion

This study represents the first comprehensive and prospective comparison of various outcomes, focusing on anorectal function in cT1‐2N0 tumors located 4–5 cm from the anal verge. Our findings indicated no significant differences between the two approaches in short‐term and oncological outcomes or urinary or male sexual function, suggesting the potential superiority of anal function in stapled anastomosis.

Regarding post‐operative complications, our study found that the rates of early anastomotic leakage within 1 month were 10.8% for hand‐sewn and 8.6% for stapled anastomosis. Additionally, anastomotic stenosis as a late complication was observed in 3.1% and 4.3% of the cases, respectively. Previous large‐scale studies have reported anastomotic leakage rates of approximately 9% and 10%, with anastomotic stenosis rates ranging from approximately 2%–11% and 4% [8, 18, 19], respectively, corroborating our findings.

Regarding anal function, our prospective comparison using Wexner scores consistently showed 2–3 points higher in the hand‐sewn than in the stapled group at 3, 6, 12, 24, and 36 months, suggesting poorer anal function in the hand‐sewn than in the stapled group. Shen et al. compared the Wexner scores after hand‐sewn and stapled anastomoses within 4 cm from the anal verge and concluded that at the 3‐year post‐operative point, the hand‐sewn group had a significantly higher Wexner score (2.6 points) than the stapled group, due to differences in gas and solid stool incontinence [20], consistent with our findings. Other studies have also indicated inferior anal function following hand‐sewn compared with stapled anastomosis. Matsunaga et al. compared anal function after hand‐sewn and stapled procedures for tumors located 4–6 cm from the anal verge at 3, 6, 12, and 24 months postoperatively and reported worse Wexner scores for hand‐sewn than for stapled cases at all time points [10]. Furthermore, Chong et al. compared anal function after hand‐sewn and stapled procedures for tumors within 5 cm from the anal verge at 6 and 36 months postoperatively and noted a higher proportion of anal dysfunction in the hand‐sewn than in the stapled group at both time points [21].

Anorectal dysfunction has a complex pathophysiology, combined with anal sphincter damage contributing to decreased sphincter pressure, reduced neo‐rectal capacity and compliance, and altered neuromuscular coordination, such as the rectoanal inhibitory reflex, which leads to the loss of anal sampling mechanisms to differentiate and control solids, liquids, and gas [22, 23, 24]. The rectoanal inhibitory reflex is a transient internal sphincter relaxation following rectal retention, which is essential for anal sampling mechanism [25]. The rectoanal inhibitory reflex is presumed to be more strongly impaired in hand‐sewn, which involves resection of the internal anal sphincter, than in stapled anastomoses. Moreover, the maximum squeezing pressure of the internal anal sphincter is influenced by the extent of sphincter resection [26]. These mechanisms support our finding that hand‐sewn anastomosis results in a worsening of Wexner scores compared with stapled anastomosis.

Regarding the recovery of anorectal function, our results showed gradual improvement over time in both groups at 1 year postoperatively, with further gradual improvements observed at 2 and 3 years postoperatively. A few studies have longitudinally investigated the anal function in both groups over a 3 years. Matsunaga et al. followed the anal function in both groups for up to 2 years postoperatively and reported a gradual decline in Wexner scores over a 2‐year period for both groups [10]. However, Kawada et al. followed post‐operative recovery in both groups for up to 2 years, noting that while stapling reached a plateau at 1 year postoperatively, hand‐sewn anastomosis showed gradual recovery up to 2 years [27]. The rectoanal inhibitory reflex recovers postoperatively, with further recovery demonstrated by the second post‐operative year compared to the first year [25], suggesting a potential reason for the continued improvement in anal function even years after hand‐sewn anastomosis.

Here, no differences were found in urinary and sexual functions between the two groups. Both functions are affected by intraoperative damage to the pelvic autonomic nervous system, consistent with the comparable outcomes observed in both groups, given their similarity in total mesorectal excision.

In terms of oncological outcomes, the theoretical background for differences between both approaches lies in the possibility of insufficient distal margin in the stapled group and the risk of tumor cell dissemination in the hand‐sewn group under inadequate surgical procedures due to communication between the bowel lumen and abdominal cavity. The distal margin was longer in the hand‐sewn (18.3 mm) than in the stapled group (12.8 mm). The ring specimen margin was removed during anastomosis. Previous studies have established a distal margin of 10 mm or more as a condition for sphincter‐preserving surgery in ultralow rectal cancer [21, 28], which was met in the stapled group. Regarding tumor dissemination in the hand‐sewn group, we presumed that the secure closure of the oral side with sufficient irrigation during the perineal procedure prevented dissemination. In this study, the 3‐year cumulative local recurrence rates for both groups were similar at 4.7% and 4.8%. Consistent with previous reports from specialized institute [11, 29, 30], no significant difference in local recurrence rates was observed between hand‐sewn and stapled anastomoses. Furthermore, no differences were found in overall and recurrence‐free survival rates in this study, consistent with previous findings. Notably, according to a nationwide study conducted across 127 variable facilities in Japan [8], the rates of local recurrence following hand‐sewn surgery for pT1 and pT2 cases were 4.2% and 8.5%, respectively, significantly higher than those for stapled anastomosis. To maintain satisfactory outcomes for hand‐sewn anastomosis and intersphincteric dissection, sufficient proficiency in patient selection and surgical techniques is required.

This study has a few limitations. First, the sample size was small with 135 cases (65 hand‐sewn and 70 stapled), which may limit the generalizability of the findings and increase the risk of sample bias. Additionally, the study lacked randomization and matching between the two groups. Consequently, there was a slight statistical difference in the distance from the anal verge to the tumor (48.7 vs. 49.5 mm). However, a difference of approximately 1 mm is unlikely to have significant clinical implications. Finally, the timing of anorectal function assessment differed between the two groups, largely due to a higher proportion of diverting stomas in the hand‐sewn group. Although we performed a subgroup analysis limited to patients who underwent ileostomy to reduce this bias, a significant difference in the time to initial evaluation remained and may have influenced the observed outcomes.

In conclusion, our findings suggest that compared with hand‐sewn anastomosis, stapled anastomosis may lead to better anorectal function while maintaining comparable safety and oncological outcomes. Therefore, stapled anastomosis may be the preferred surgical approach, particularly in cases where oncological safety is secured.

## Author Contributions

**Masakatsu Numata:** conceptualization, investigation, methodology, writing – original draft. **Jun Watanabe:** conceptualization, investigation, methodology, validation, supervision. **Yuichiro Tsukada:** conceptualization, investigation, methodology, validation, supervision. **Yusuke Suwa:** data curation. **Yosuke Fukunaga:** data curation. **Yasumitsu Hirano:** data curation. **Kazuhiro Sakamoto:** data curation. **Hiroki Hamamoto:** data curation. **Masanori Yoshimitsu:** data curation. **Hisanaga Horie:** data curation. **Nobuhisa Matsuhashi:** data curation. **Yoshiaki Kuriu:** data curation. **Shuntaro Nagai:** data curation. **Madoka Hamada:** data curation. **Shinichi Yoshioka:** data curation. **Shinobu Ohnuma:** data curation. **Tamuro Hayama:** data curation. **Koki Otsuka:** data curation. **Yusuke Inoue:** data curation. **Kazuki Ueda:** data curation. **Yuji Toiyama:** data curation. **Satoshi Maruyama:** data curation. **Shigeki Yamaguchi:** data curation. **Keitaro Tanaka:** data curation. **Takeshi Naitoh:** supervision. **Masahiko Watanabe:** supervision. **Motoko Suzuki:** formal analysis. **Toshihiro Misumi:** supervision. **Masaaki Ito:** conceptualization, investigation, methodology, validation, supervision.

## Ethics Statement

The study protocol was approved by the Ethics Committee of the Japanese Society for Cancer of the Colon and Rectum and was sanctioned and monitored by the institutional review board of each participating hospital.

## Consent

All the participants provided written informed consent.

## Conflicts of Interest

The authors Jun Watanabe and Yuji Toiyama are an editorial member of the *Annals of Gastroenterological Surgery*. The other authors declare no conflicts of interest.

## Supporting information


**Figure S1.** Wexner score change from the baseline (patients with splenic flexure mobilization).

**Figure S2.** Wexner score change from the baseline (patients without splenic flexure mobilization).

**Figure S3.** Wexner score change from the baseline (patients with diverting ileostomy).

**Table S1.** Backgrounds for patients with splenic flexure mobilization.

**Table S2.** Backgrounds for patients without splenic flexure mobilization.
