# Supplementary figures and images for: Patient‐Reported Outcomes and Surgical Results of Hand‐Sewn Versus Stapled Anastomosis for Lower Rectal Cancer Located 4–5 cm From the Anal Verge: A Subanalysis of the Ultimate Study
Source: Ann Gastroenterol Surg. 2025 Jul 9;9(6):1215–24. doi: 10.1002/ags3.70063 (PMC12586937; doi:10.1002/ags3.70063)

Supplementary Figure 1: Wexner Score Change from Base Line  
(With Splenic Flexure Mobilization)

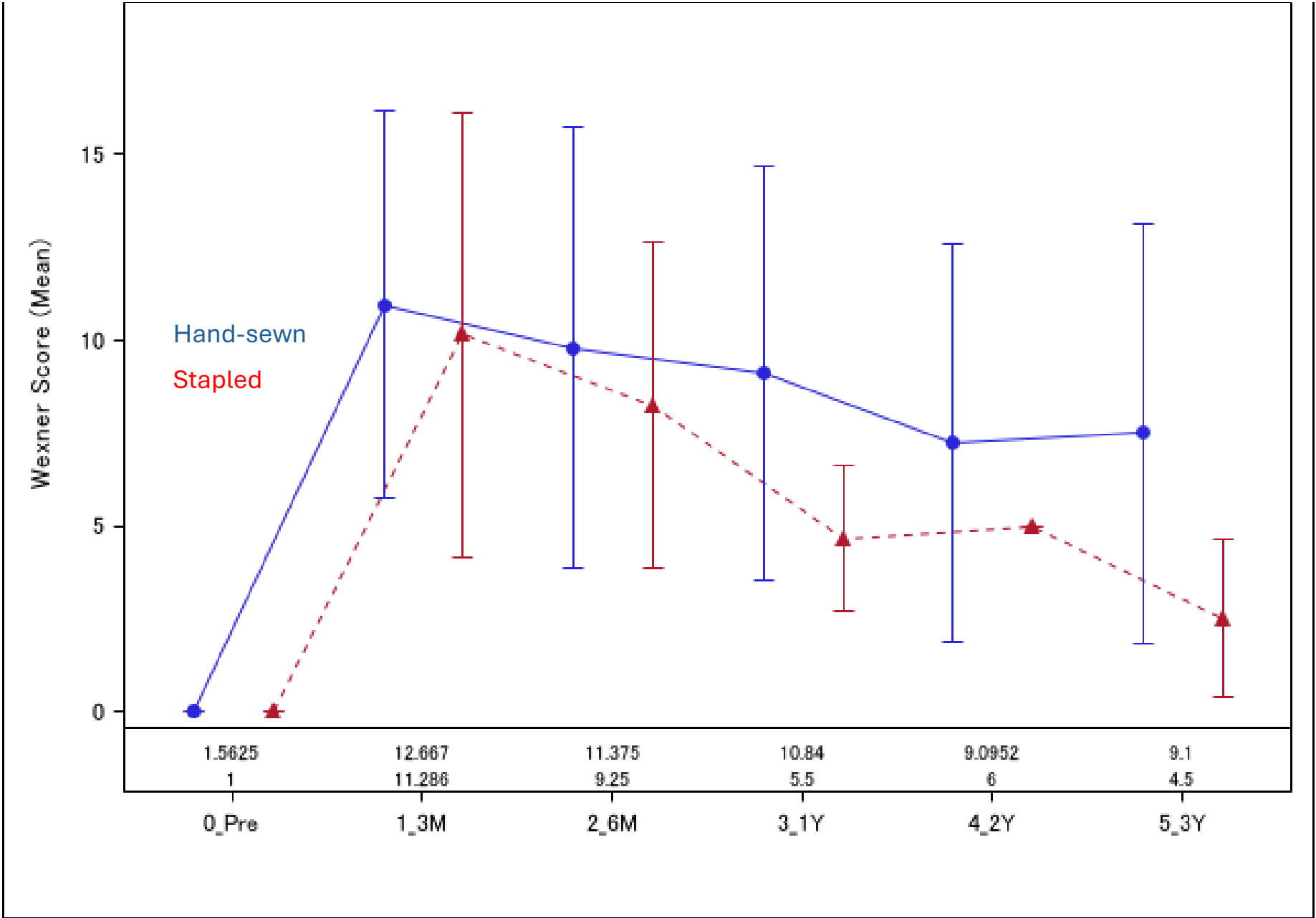

Supplement: Supplementary file 1 — Figure S1. Wexner score change from the baseline (patients with splenic flexure mobilization). [file AGS3-9-1215-s005.pdf]

Supplementary Figure 3: Wexner Score Change from Base Line  
(Without Diverting Ileostomy)

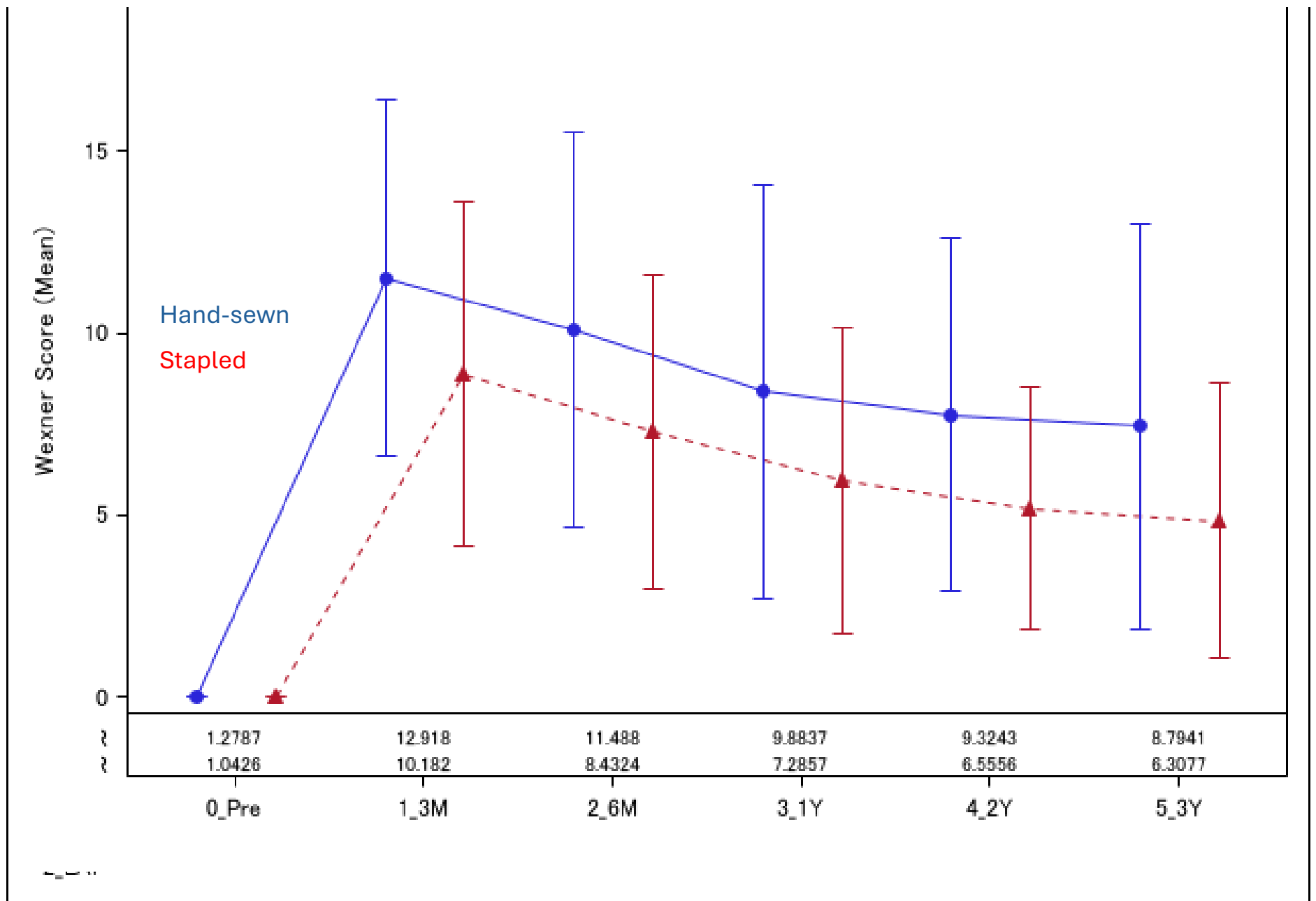

Supplement: Supplementary file 3 — Figure S3. Wexner score change from the baseline (patients with diverting ileostomy). [file AGS3-9-1215-s002.pdf]
