# Supplementary material for: Patient‐Reported Outcomes and Surgical Results of Hand‐Sewn Versus Stapled Anastomosis for Lower Rectal Cancer Located 4–5 cm From the Anal Verge: A Subanalysis of the Ultimate Study
Source: Ann Gastroenterol Surg. 2025 Jul 9;9(6):1215–24. doi: 10.1002/ags3.70063 (PMC12586937; doi:10.1002/ags3.70063)

Supplementary Figure 2: Wexner Score Change from Base Line (Without Splenic Flexure Mobilization)

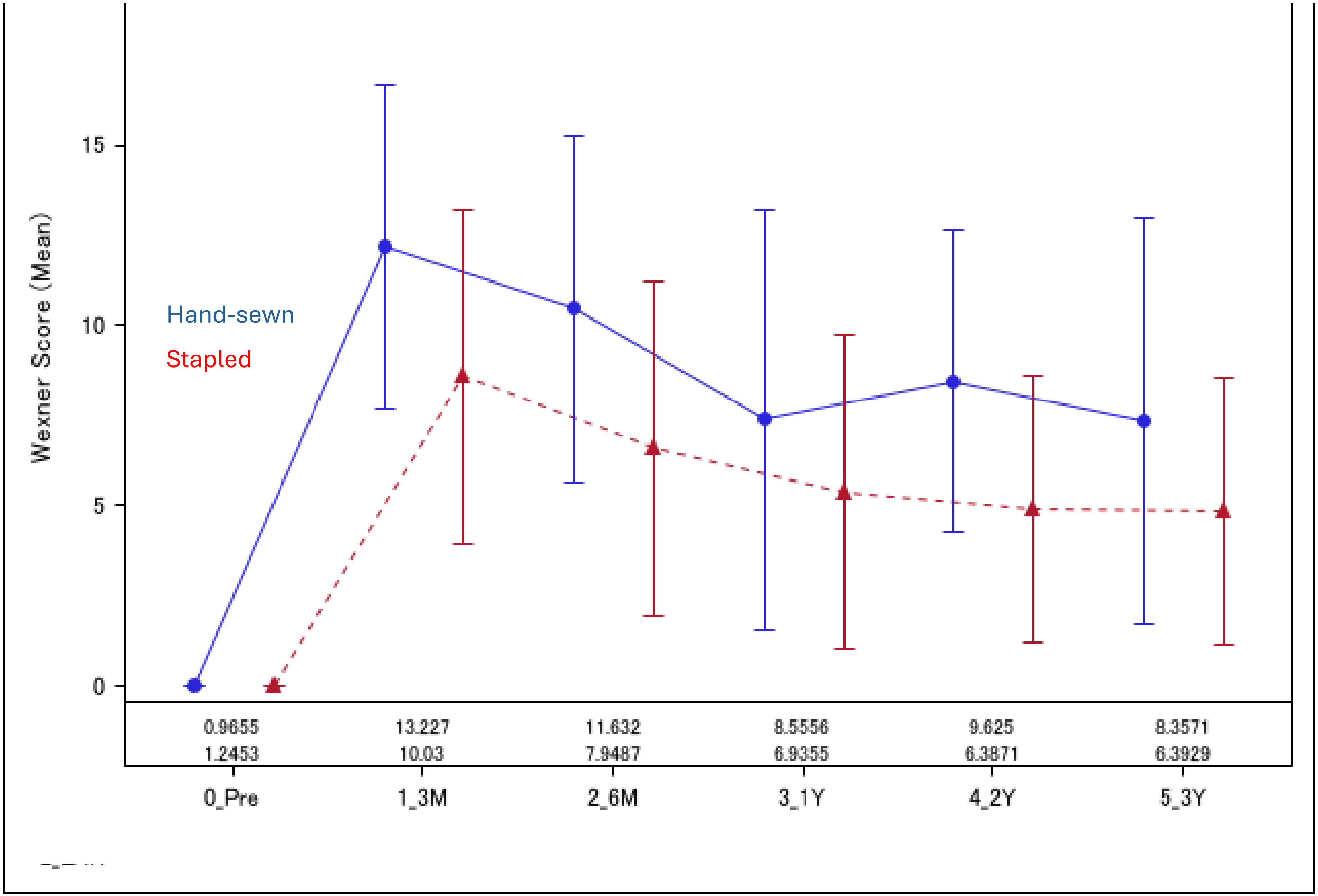

Supplement: Supplementary file 2 — Figure S2. Wexner score change from the baseline (patients without splenic flexure mobilization). [file AGS3-9-1215-s003.pdf]
