# Supplementary material for: Patient‐Reported Outcomes and Surgical Results of Hand‐Sewn Versus Stapled Anastomosis for Lower Rectal Cancer Located 4–5 cm From the Anal Verge: A Subanalysis of the Ultimate Study
Source: Ann Gastroenterol Surg. 2025 Jul 9;9(6):1215–24. doi: 10.1002/ags3.70063 (PMC12586937; doi:10.1002/ags3.70063)
Supplement: Supplementary file 4 — Table S1. Backgrounds for patients with splenic flexure mobilization. [file AGS3-9-1215-s001.docx]

**Supplementary Table 1**

**Backgrounds for Patients with Splenic Flexure Mobilization**

|  |  | **Hand-sewn**  **(n=33)** | **Stapled**  **(n=10)** | **P** |
| --- | --- | --- | --- | --- |
| **Age** |  | 64.0 (9.13) | 64.7 (11.49) | 0.49 |
| **Sex** | **Male** | 28 (84.8%) | 9 (90.0%) | 0.68 |
|  | **Female** | 5 (15.2%) | 1 (10.0%) |  |
| **ECOG-PS**^a^ | **0** | 33 (100.0%) | 10 (100.0%) |  |
| **Abdominal Surgery History** |  | 6 (18.2%) | 1 (10.0%) | 0.53 |
| **BMI**^b^ |  | 23.1 (3.24) | 22.8 (3.79) | 0.77 |
| **Tumor distance from AV**^c^ |  | 48.9 (4.64) | 50.0 (0.00) | 0.27 |
| **Tumor distance from DL**^d^ |  | 30.5 (6.66) | 30.0 (0.00) | 0.83 |
| **CEA**^e^ |  | 2.9 (1.87) | 2.2 (1.38) | 0.48 |
| **CA19-9**^f^ |  | 11.7 (11.77) | 9.0 (7.38) | 0.11 |
| **Tumor laterality** | **Left** | 5 (15.2%) | 1 (10.0%) | 0.11 |
|  | **Right** | 3 (9.1%) | 4 (40.0%) |  |
|  | **Anterior** | 15 (45.5%) | 2 (20.0%) |  |
|  | **Posterior** | 10 (30.3%) | 3 (30.0%) |  |
| **cT** | **T1** | 21 (63.6%) | 3 (30.0%) | 0.06 |
|  | **T2** | 12 (36.4%) | 7 (70.0%) |  |
| **cN** | **N0** | 33 (100) | 10 (100) |  |
| **Adjuvant chemotherapy** |  | 6 (18.2%) | 1 (10.0%) | 0.53 |

a: ECOG-PS: Eastern Cooperative Oncology Group Performance Status

b: BMI: Body mass index

c: AV: Anal verge

d: DL: Dentate line

e: CEA: Carcinoembryonic antigen

f: CA19-9: Carbohydrate antigen 19-9
