# Supplementary material for: Patient‐Reported Outcomes and Surgical Results of Hand‐Sewn Versus Stapled Anastomosis for Lower Rectal Cancer Located 4–5 cm From the Anal Verge: A Subanalysis of the Ultimate Study
Source: Ann Gastroenterol Surg. 2025 Jul 9;9(6):1215–24. doi: 10.1002/ags3.70063 (PMC12586937; doi:10.1002/ags3.70063)
Supplement: Supplementary file 5 — Table S2. Backgrounds for patients without splenic flexure mobilization. [file AGS3-9-1215-s004.docx]

**Supplementary Table 2**

**Backgrounds for Patients without Splenic Flexure Mobilization**

|  |  | **Hand-sewn**  **(n=32)** | **Stapled**  **(n=60)** | **P** |
| --- | --- | --- | --- | --- |
| **Age** |  | 63.2 (12.12) | 61.9 (10.23) | 0.35 |
| **Sex** | **Male** | 22 (68.8%) | 36 (60.0%) | 0.40 |
|  | **Female** | 10 (31.3%) | 24 (40.0%) |  |
| **ECOG-PS**^a^ | **0** | 28 (87.5%) | 57 (95.0%) | 0.19 |
|  | **1** | 4 (12.5%) | 3 (5.0%) |  |
| **Abdominal Surgery History** |  | 5 (15.6%) | 19 (31.7%) | 0.09 |
| **BMI**^b^ |  | 22.8 (2.62) | 23.3 (3.42) | 0.68 |
| **Tumor distance from AV**^c^ |  | 48.4 (2.35) | 49.4 (1.80) | 0.06 |
| **Tumor distance from DL**^d^ |  | 27.8 (4.57) | 28.3 (5.19) | 0.41 |
| **CEA**^e^ |  | 2.8 (2.66) | 2.8 (3.21) | 0.48 |
| **CA19-9**^f^ |  | 9.9 (8.88) | 9.2 (7.47) | 0.81 |
| **Tumor laterality** | **Left** | 6 (18.8%) | 11 (18.3%) | 0.53 |
|  | **Right** | 3 (9.4%) | 7 (11.7%) |  |
|  | **Anterior** | 13 (40.6%) | 16 (26.7%) |  |
|  | **Posterior** | 10 (31.3%) | 26 (43.3%) |  |
| **cT** | **T1** | 14 (43.8%) | 42 (70.0%) | 0.01 |
|  | **T2** | 18 (56.3%) | 18 (30.0%) |  |
| **cN** | **N0** | 32 (100) | 60 (100) |  |
| **Adjuvant chemotherapy** |  | 7 (21.9%) | 9 (15.0%) | 0.40 |

a: ECOG-PS: Eastern Cooperative Oncology Group Performance Status

b: BMI: Body mass index

c: AV: Anal verge

d: DL: Dentate line

e: CEA: Carcinoembryonic antigen

f: CA19-9: Carbohydrate antigen 19-9
